# Biologic monotherapy in the biologic naïve patient with rheumatoid arthritis (RA): results from an observational study

**DOI:** 10.1007/s00296-020-04531-6

**Published:** 2020-02-21

**Authors:** Rosalind Benson, Sizheng Steven Zhao, Nicola Goodson, Rikki Abernethy, Devesh Mewar, Theresa Barnes

**Affiliations:** 1 Liverpool University Hospitals NHS Foundation Trust, Royal Liverpool University Hospital, Prescot Street, Liverpool, L7 8XP UK; 2 Liverpool University Hospitals NHS Foundation Trust, Aintree University Hospital, Lower Lane, Liverpool, L9 7AL UK; 3 St Helens and Knowsley Teaching Hospitals NHS Trust, Warrington Rd, Prescot, L35 5DR UK; 4 Department of Rheumatology, Countess of Chester Hospital, Liverpool Rd, Chester, CH2 1UL UK

**Keywords:** Rheumatoid arthritis, Biologic therapies, DMARDs, Immunotherapy

## Abstract

Approximately one-third of patients on biologic therapy for rheumatoid arthritis (RA) receive them as monotherapy. There are few head-to-head randomised control trials comparing biologics as monotherapy. Our aim was to compare the efficacy and persistence of multimodal biologic agents as monotherapy in biologic naïve patients with RA in the real-world setting. A multicentre retrospective observational study was carried out comparing TNF inhibitors (TNFi), IL6 receptor inhibitor (IL6Ri) and CTLA-4 inhibitor (CTLA-4i) monotherapy in biologic naïve RA patients. The primary study outcome was DAS28 score at 6, 12, and 18 months. 126 patients were enrolled; 98 patients (78%) were taking TNFi, 19 patients (15%) IL6Ri and 10 (8%) CTLA-4i with similar baseline characteristics of sex and age across groups. Patients in the CTLA-4i group were more often seropositive and had greater numbers of comorbidities. At 6 and 12 months, patients in the IL6Ri group had a lower DAS28 score compared to TNFi monotherapy. Those on CTLA-4i monotherapy also had a lower DAS28 score at 6 months than the TNFi group, although differences were lost by 12 months. Drug retention at 18 months was highest in the IL6Ri arm (68%) and CTLA-4i arm (80%) compared with only 55% in the TNFi group. Our findings support current guidance that IL6Ri should be considered in biologic naïve patients requiring biologic monotherapy, but also indicated that CTLA-4i could be an option.

## Introduction

Up to a third of rheumatoid arthritis (RA) patients on biologic therapy do not take concurrent conventional synthetic disease-modifying antirheumatic drugs (csDMARDs) contrary to the recommendations from the most recent EULAR guidance. [1, 2] There are many reasons for this including adverse effects, poor drug adherence and contraindications to csDMARDs [3]. Despite this commonplace real-world practice, there are few head-to-head randomised control trials (RCTs) comparing biologic monotherapies to guide our prescribing and those published have limited follow-up periods of typically 24 weeks [3]. The ADACTA and MONARCH trials showed superiority of IL6 receptor inhibitor (IL6Ri) monotherapy to adalimumab (a TNFi) monotherapy in reduction of DAS28 score at 24 weeks [4, 5]. Furthermore, large commercial RCTs frequently exclude patients with complex comorbidity, thus results are not always directly applicable in real-world settings. Aiming to address this knowledge gap, we compared the efficacy and persistence of multimodal biologic agents as monotherapy in biologic naïve patients with RA in the real-world setting focusing specifically on the use of TNFi, IL6Ris and CTLA-4is.

## Patients and methods

We conducted a retrospective observational study across four UK Rheumatology units. Ethical approval for the study protocol was sought and gained with the UK Health Research Authority following research ethics committee review. Data were extracted from clinical biologic drug monitoring databases at each participating unit. Data were anonymised prior to analysis. All patients with a diagnosis of RA (as defined by their rheumatologist) initiating a biologic therapy without concurrent csDMARD therapy between 2012 and 2016 were included. In line with NHS prescribing policies, to be applicable for funding for biologic monotherapy, all patients were either intolerant or had contraindications to csDMARDs. Only subcutaneous biologic agents were included. DAS28 scores had to be greater than 5.1 on two occasions to be applicable for funding through the National Health Service (NHS) for biologic therapy. To continue to receive NHS funding for biologic therapy, patients must show initial and continued response to treatment as demonstrated by a reduction in DAS28 score. Patients are assessed routinely at baseline and then at every 6 months thereafter to comply with national guidance. Due to the recognised effect of IL6-i on CRP, we only included DAS28 score calculated using the ESR.

Patients on concurrent csDMARDs, long-term steroids or previous bDMARD exposure were excluded. During the study, some patients may have received short courses of oral steroids or intraarticular steroids if they had a flare of disease. Due to the ad hoc nature of these prescriptions, it was not possible to collect these data. However, at a patient’s 6-month review, routine questioning identifying repeated disease flares would be an indication of failure of biologic treatment prompting change in treatment which would be recorded.

Baseline demographical data was collected including age, sex, seropositivity status using rheumatoid factor (RF) or presence of anti-citrullinated protein antibodies (ACPA) where available and presence of comorbidities (specifically including malignancy, diverticulitis, severe infection, heart failure, ischaemic heart disease, demyelinating disease, tuberculosis, diabetes or interstitial lung disease). Rationale for prescription of biologic agent as monotherapy was also recorded.

We compared biologic classes TNFi, IL6Ri and CTLA-4 inhibitors. The primary outcome was absolute DAS28 at 6, 12 and 18 months.

Covariates were pre-defined according to their potential association with the outcome, including age, sex, seropositivity status using ACPA or RF, comorbidities (including malignancy, demyelinating disease diverticulitis, severe infection, heart failure, ischaemic heart disease, tuberculosis, diabetes or interstitial lung disease), previous DMARD history and the reason for discontinuation; baseline DAS28-ESR was recorded with individual components. Drug exposure duration was recorded.

### Statistical analyses

Comparison of patient and disease characteristics between the treatment groups was performed using ANOVA and Kruskal–Wallis test for normal and non-normally distributed variables and Chi-squared or Fisher’s exact test for categorical variables.

At each time point, we used weighted logistic regression with drug group as the only independent variable (TNFi as the reference) and each outcome measure in turn as the dependent variable. Covariates were balanced using matching weights. Multigroup propensity score (PS) matching excludes many individuals and is computationally demanding. In our case, where each group size is relatively small, matching would be prohibitive for analysis. Matching weights are an extension of inverse probability of treatment weighting (IPTW), which emulates a PS-matched population. This method retains all individuals in the analysis and thus the advantage of superior mean squared error, particularly in the case of unequal sized groups and poor covariate overlap [6]. Briefly, it replaces the IPTW numerator with the smallest PS of each treatment group. A multinomial logistic model was used to construct IPTW for each treatment group. Independent variables for the IPTW model included all baseline covariates as well as all baseline outcome measures.

Missing outcomes at each time point are unlikely to be random thus leading to bias. We therefore used inverse probability of censoring weights (IPCW) to make missingness random with respect to baseline variables, as follows: the numerator is the predicted probability from logistic models of not being missing conditioned on treatment group, the denominator additionally conditioned on covariates. To generate the above weights, multinomial and logistic models required complete data for all covariates. Multiple imputation was performed using chained equations (-mi impute mice- command in Stata v14). All variables in each IPW model were included in the respective imputation models, with 30 imputed datasets.

## Results

### Patient characteristics

We recruited 126 patients. Reasons for prescriptions of biologic agents as monotherapy included previous adverse reactions to csDMARDs resulting in gastrointestinal side effects, rashes, blood dyscrasias or hepatotoxicity, while for other patients csDMARDs were contraindicated.

Baseline characteristics are shown in Table 1. 98 patients (78%) were taking TNFi, this group comprised etanercept (76 patients), adalimumab (10 patients), certolizumab (5 patients) and golimumab (7 patients). 19 patients (15%) were on an IL6Ri and 10 (8%) CTLA-4i. Of those on TNFi, the proportion of females on each biologic and patient age was similar across all drugs. Patients starting IL6Ri had significantly shorter disease duration. A greater proportion of patients on CTLA-4i and TNFi had more comorbidities than TNFi (Table 2) and those in the CTLA-4i were more likely to have multiple comorbidities at baseline. Those on CTLA-4i were more likely to be seropositive. Baseline disease activity according to DAS28 and its components were generally comparable.

**Table 1:** Baseline characteristics

*n*	All TNFi	IL6Ri	CTLA-4i	*p* value
	98	19	10	
Mean age, years (SD)	60.9 ( 12.0)	62.4 (12.4)	62.3 ( 11.4)	0.84
Female, *n* (%)	77 (79%)	12 (63%)	7 (70%)	0.33
Disease duration, *n* (%)				
1–2 years	1 (1%)	4 (21%)	1 (10%)	0.001
3–4 years	18 (18%)	6 (32%)	1 (10%)	
≥ 5 years	79 (81%)	9 (47%)	8 (80%)	
RF positive*, *n* (%)	36 (59%)	4 (50%)	4 (80%)	0.590
ACPA positive*, *n* (%)	50 (72%)	6 (67%)	5 (100%)	0.417
RF or ACPA seropositive*, *n* (%)	68 (69%)	9 (47%)	9 (90%)	0.065
Mean number of comorbidities (SD)	0.3 (0.7)	0.8 (1.2)	1.4 (1.6)	< 0.001
DAS28-ESR, mean (SD)	6.1 (0.9)	6.3 ( 0.9)	6.3 (0.8)	0.50
Tender joint count, median (IQR)	12.0 (8.0, 18.0)	13.0 (9.0, 21.0)	14.0 (11.0, 15.0)	0.45
Swollen joint count, median (IQR)	6.0 (3.0, 9.0)	5.0 (4.0, 8.0)	5.5 (3.0, 7.0)	0.61
Median ESR, mm/hr (IQR)	33.0 (19.0, 52.0)	34.0 (17.0, 76.0)	34.0 (12.0, 98.0)	0.71
Global VAS, mean (SD)	78.2 (19.3)	79.6 (19.4)	82.6 (15.1)	0.77

^*^Data incomplete for autoantibodies

*TNFi* TNF inhibitor, *IL6Ri* IL6 receptor inhibitor, *CTLA-4i* CTLA-4 inhibitor, *RF* rheumatoid factor, *ACPA* anti-citrullinated protein antibodies, *VAS* visual analogue scale (0–100 mm)

**Table 2:** Comorbidities at baseline

	TNFi	IL6Ri	CTLA-4i
Number with available data	55	14	8
Malignancy	0	1	1
Demyelinating disease	0	0	0
Diverticulitis	3	1	0
Cardiac failure	0	0	0
Atherosclerotic disease	5	2	0
TB	2	0	0
Diabetes	1	1	2
Interstital lung disease	0	2	1
Other	3	3	5

### Comparison of DAS28 scores across drug groups

Compared to patients on TNFi at 6 months, DAS28 was lower for patients on IL6Ri by 1.84 units (95% CI − 2.57, − 1.11) and CTLA-4i by 0.33 units (95% CI − 0.60, − 1.25). At 12 months, DAS28 was lower in patients on IL6Ri [0.90 units (95% CI − 1.90, 0.13)] and CTLA-4i [0.24 units (95% CI − 1.46, 0.97)] compared to TNFi. At 18 months, those on IL6Ri scored 0.90 units lower (95% CI − 1.90, 0.13) and CTLA-4i 0.80 units higher (95% CI 0.09, 1.53) than TNFi. IL6Ri led to a striking reduction in ESR compared to TNFi at all time points. At both 12 months and 18 months, IL6i and CTLA-4i led to significantly fewer swollen joints than those on TNFi (Table 3).

**Table 3:** DAS28 and components thereof compared against TNF inhibitors at each time point

		6 months		12 months		18 months	
		Coefficient (95% CI)	*P* value	Coefficient (95% CI)	*P* value	Coefficient (95% CI)	*P* value
IL6Ri	DAS28	− 1.84 (− 2.57, − 1.11)	< 0.001	− 1.69 (− 2.30, − 1.07)	< 0.001	− 0.88 (− 1.90, 0.31)	0.087
	SJC	− 0.88 (− 1.95, 0.19)	0.110	− 1.25 (− 1.93, − 0.57)	0.001	− 1.85 (− 3.48, − 0.22)	0.030
	TJC	− 2.11 (− 4.46, 0.22)	0.080	− 1.48 (− 2.90, − 0.07)	0.040	0.25 (− 0.39, 0.88)	0.440
	ESR	− 22.00 (− 27.35, − 17.60)	< 0.001	− 15.00 (− 22.01, − 7.92)	< 0.001	− 15.93 (− 32.04, 0.18)	0.053
	VAS	− 8.50 (26.70, 8.70)	0.330	− 13.70 (− 35.36, 7.96)	0.210	− 6.89 (− 20.72, 6.94)	0.330
CTLA-4i	DAS28	− 0.33 (− 0.60, 1.25)	0.480	− 0.24 (− 1.50, 0.97)	0.690	0.81 (0.09, 1.53)	0.030
	SJC	− 0.79 (− 1.97, − 0.40)	0.190	− 0.76 (− 1.83, 0.32	0.170	− 1.57 (− 3.00, − 0.18)	0.030
	TJC	5.98 (− 3.21, 15.16)	0.200	1.65 (− 2.56, 5.86)	0.440	2.59 (− 0.26, 5.37)	0.080
	ESR	− 13.09 (− 25.55, − 0.63)	0.040	11.63 (− 25.67, 48.94)	0.540	− 3.02 (− 39.82, 33.78)	0.870
	VAS	− 4.22 (− 15.28, 6.82)	0.450	− 12.85 (− 34.52, 8.83)	0.240	25.49 (− 4.03, 55.00)	0.090

*IL6Ri* IL6 receptor inhibitor, *CTLA-4i* CTLA-4 inhibitor, *SJC* swollen joint count, *TJC* tender joint count, *ESR* (mm/hr), *VAS* Patient Global Visual Analogue scale

More patients in the IL6Ri and CTLA-4i groups reached EULAR DAS28 defined remission at 18 months (54% and 50%, respectively) compared with only 39% in the TNFi group. The percentage of patients reaching low disease activity (DAS28 < 3.2) at 18 months in the IL6Ri group was higher (85%) compared with 63% in both other drug groups. These results may explain why drug retention was greater in the non-TNFi groups at 18 months: 68% in the IL6Ri group and 80% in CTLA-4i versus 55% in the TNFi group. At the end of follow-up, inefficacy was the reason for discontinuation of biologic therapy in 18% of patients on TNFi compared with only 5% on IL6Ri and 10% on a CTLA-4i. Adverse reactions caused cessation of biologic treatment in 13% of patients initiated on TNFi, whereas in the IL6Ri it was 21% and the CTLA-4i, 30%.

## Discussion

In this study using real-world data, IL6Ri led to lower DAS28 at 6 and 12 months compared to TNFi monotherapy, although differences were attenuated by 18 months. Similarly, CTLA-4i monotherapy resulted in lower DAS28 at 6 months than TNFi, although superiority was lost by 12 months. Drug persistence at 18 months was great in the CTLA-4i and IL6Ri groups than those on TNFi.

To date, there is only one head-to-head RCT of CTLA-4i with the TNFi adalimumab, however, all patients were on concurrent methotrexate [7]. Our study demonstrated significant reduction of SJC at 18 months and comparably low DAS28 score for those on CTLA-4i versus TNFi. Notably, those on CTLA-4i had more comorbidities (a typically more challenging group to treat), the high drug retention of 80% at 18 months would support the use of CTLA-4i in this cohort. These real-world data are particularly useful as the BSR biologics registry does not include CTLA-4i monotherapy so there is little published data on this cohort. The observational study by Jorgensen et al. compared biologic monotherapy (including those on TNFi, CTLA-4i and IL6Ri) and disease activity at 6 months only [8]. The study did not adjust for potential confounding factors. It included patients both newly started on the drug during the study period and those started on it prior to study initiation, although post hoc statistical analysis found there was no difference in drug adherence between groups or CDAI remission rates. Furthermore, 22% of all patients included were on long-term prednisolone complicating the analysis. Like the study by Bachkaus et al. (which compared TNFi and Tocilizumab mono therapy), Jorgensen et al. included patients who had been on multiple biologic therapies previously which could indicate harder-to-treat disease [9]. In a further study by Jorgenson et al., no adjustment for potential confounders has been included therefore the results are open to confounding by indication [10].

By contrast, our study design including only biologic naive patients and adjustment for potential confounding factors including comorbidities, seropositivity and age amongst others makes the results more easily interpretable. Due to the study design relying on retrospective data collection, there were some data points missing particularly in regard to seropositivity status of patients, however, our statistical analysis took account of this.

Key strengths of this study included examination of DAS28 components. We showed that IL6Ri had profound impact on ESR, as expected, but also a lower SJC for IL6i and CTLA-4i monotherapies. Our duration of follow-up was also longer than typical RCTs, allowing us to see CTLA-4i may be more efficacious initially, but not at long-term follow-up. Our study was limited by the lack of other outcomes including CRP. Patients enrolled in this study had high disease activity with baseline median DAS28 scores 6.1–6.3 across the drug groups. We recognise that in some clinical contexts, patients maybe commenced on biologic monotherapy with less active disease. Furthermore, caution should be taken drawing conclusions from our small sample size in particular relating to the IL6i and CTLA-4i groups. However, the effect size of TNFi vs IL6Ri comparison was similar to randomised trials [4].

## Conclusion

Our findings support EULAR guidance that IL6Ri should be considered as a first-line biologic in the biologic naive patient requiring monotherapy [2]. However, CTLA-4i may also have a role in this patient population and further studies of larger patient numbers are needed to investigate this further.
